# Plasma CXCL3 Levels Are Associated with Tumor Progression and an Unfavorable Colorectal Cancer Prognosis

**DOI:** 10.1155/2022/1336509

**Published:** 2022-05-26

**Authors:** Can Cui, Rui Zhang, Feng Gu, Yunfeng Pei, Li Sun, Yueyang Huang, Guoping Niu, Jian Li

**Affiliations:** Department of Clinical Laboratory, Xuzhou Central Hospital, China

## Abstract

**Background:**

The CXC chemokines belong to a unique family of chemotactic cytokines that influence the initiation, progression, and clinical outcome of many tumor types. Herein, we investigated the association of the CXC-chemokine ligand 3 (CXCL3) with tumor progression and an unfavorable prognosis for colorectal cancer (CRC).

**Methods:**

The quantitative real-time polymerase chain reaction was used to explore the expression of CXCL3 in CRC tissue, adjacent tissue, and plasma. The usefulness of plasma levels of CXCL3 for the diagnosis of CRC was evaluated by receiver operating characteristic curve analysis. Pearson's correlation analysis assessed relationships among plasma CXCL3, cancer tissue CXCL3, and plasma carcinoembryonic antigen (CEA). Kaplan–Meier analysis was used to assess the survival of CRC patients with high and low expression levels of CXCL3. Survival differences were compared by log-rank test.

**Results:**

Initial analysis of the GSE156720 dataset identified CXCL3 as the most enriched CXCL gene in CRC patients. Higher CXCL3 levels were detected in CRC tissue than in adjacent tissue (*P* < 0.001). Compared to healthy controls, CRC patient plasma CXCL3 levels were higher (*P* < 0.001). The area under the curve was 0.81 with a sensitivity of 0.71 and specificity of 0.82, distinguishing CRC from other tumor types. Plasma CXCL3 was positively related to CXCL3 in cancer tissue (*r* = 0.78, *P* < 0.01), and also to plasma CEA (*r* = 0.50, *P* < 0.01). Plasma CXCL3 was also related to tumor size (*P* = 0.034), staging (*P* < 0.001), tumor stage (*P* = 0.003), differentiation (*P* = 0.001), and lymph node metastasis (*P* = 0.007), but not to sex (*P* = 0.853), age (*P* = 0.691), tumor site (*P* = 1.347), or distant metastasis (*P* = 1.218).

**Conclusions:**

CXCL3 levels were increased in CRC patients, with plasma CXCL3 levels associated with tumor progression and an unfavorable CRC prognosis. The results of this study suggest that plasma CXCL3 may be a novel diagnostic and prognostic biomarker for CRC.

## 1. Introduction

Colorectal cancer (CRC) is one of the most common cancers worldwide, with approximately 1 million new cases diagnosed each year [1, 2]. In the past 20 years, the incidence of CRC in China has ranked fourth among all malignant tumors and fifth for mortality [3, 4]. The environment is a significant risk factor for CRC, and while the incidence of CRC can be reduced through lifestyle changes, there is a range of risk factors that cannot be changed, such as age, family, and personal history [5, 6]. Compared with other digestive tract cancers, the prognosis for rectal cancer is relatively good, but because early symptoms are not obvious, most patients are diagnosed during the middle to late disease. Consequently, treatment is often unsatisfactory, with recurrence and metastasis the leading causes of rectal cancer death [7]. As such, the identification of new diagnostic markers is essential for early cancer diagnosis and a reduction in the high CRC mortality rate. CXCs (CXCL1-17) are a class of soluble, proinflammatory, and highly conserved cytokines. CXC's are soluble cholinesterases that interact with cognate cellular receptors, stimulating cells, and inducing directed chemotaxis. Chemotaxis, migration, and adhesion play essential roles in developing various tumors [8–10]. CXCL3 encoded by the human GRO gene and located within the chromosomal region, 4q13.3. CXCL3 is a granulocyte chemoattractant (GCP-2) and is an ELR + chemokine, similar to other ELR + chemokines that, with alternative splicing, can result in multiple transcript variants. CXCL3 plays a vital role in angiogenesis, tumorigenesis, and cell invasion [11–13]. See et al. demonstrated overexpression of CXCL3 to be closely related to breast cancer metastasis [14]. Multiple studies have found CXCL3 to participate in the development of many inflammatory and autoimmune diseases and the progression and metastasis of many tumor types [15–17]. However, the function of CXCL3 in CRC patients is not well established. This study aimed to explore the expression level and clinical significance of CXCL3 in CRC patients. We found the expression of CXCL3 to be relatively high and stable in cancer tissue and the plasma of CRC patients. CXCL3 levels correlated with carcinoembryonic antigen (CEA) levels, a clinical tumor marker of CRC. Moreover, levels of CXCL3 were found to distinguish CRC from other gastrointestinal tumors.

## 2. Materials and Methods

### 2.1. Patients and Samples

This study was conducted in Xuzhou Central Hospital and was approved by the Research Ethics Committee of Xuzhou Central Hospital. A total of 59 CRC patients (42 males and 17 females, mean age 56 ± 19 years) underwent surgery at Xuzhou Central Hospital from January 2018 to June 2020. CRC cancer and adjacent tissue (5 cm removed from the cancer tissue) were excised and digested with collagenase for 15 minutes to prepare cell suspensions that were stored in liquid nitrogen. 169 patients (119 males, 50 females, mean age 55 ± 17 years) with nonoperative CRC and 219 healthy subjects (153 males, 66 females, mean age 57 ± 18 years) were also evaluated. Plasma samples were collected from all patients and control subjects. Patients had no other underlying medical history and did not receive radiotherapy, chemotherapy, or immunotherapy. All patients signed informed consent before sampling. All tissue and plasma samples were kept at -80 °C before assessment. Plasma levels of the classical tumor marker CEA (carcinoembryonic antigen) were assessed using the CMIA (chemiluminescent microparticle immunoassay) method on ARCHITECT 8200 ci (Abbott Laboratories, Abbott Park, IL, USA).

### 2.2. RNA Extraction

Total cells from tissue samples or plasma were collected and resuspended in lysis buffer (10 mM NaCl, 20 mM MgCl, ten mM Tris-HCl, pH 7.8, 5 mM DTT, 0.5% NP-40) kept in ice for 5 minutes. And then, the fraction was subjected to protease treatment for 20 minutes at 37 °C by adding an equal volume of proteinase K solution (300 mM NaCl, 0.2 M Tris-HCl, pH 7.5, 25 mM EDTA, 2% SDS, and 0.1 mg/ml proteinase K), and RNA was purified using the QIAzol Lysis Reagent (Qiagen, Germany). The RNA was subjected to DNase treatment; cDNA was synthesized using the QuantiTect Reverse Transcription Kit (Qiagen, Germany) according to the manufacturer's protocol.

### 2.3. qRT-PCR Analysis

qRT-PCR was performed using a Bio-Rad My cycle (Bio-Rad, CA, USA) according to the manufacturer's instructions. *β*-actin was used as a reference gene. The PCR primer for CXCL3 was F: 5′-CGCCCAAACCGAAGTCATAG-3′ R: 5′-GCTCCCCTTGTTCAGTATCTTTT-3′ and *β*-actin F: 5′-AATATGTGTGTCGCGGGATG-3′, R: 5′- CTCCTTAATGTCACGCACGCACGA-3′. The reaction conditions were: 95 °C, 15 s; 60 °C, 30 s; 74 °C, 30 s; and 72 °C, 20 s, with 40 cycles of amplification. All experiments were repeated at least three times. The analysis was performed using the 2^−*ΔΔ*Ct^ method, with *β*-actin as the endogenous control.

### 2.4. Statistical Analysis

Statistical analysis was conducted using GraphPad prism 8.0. The data conforming to normal distribution were expressed by mean ± standard deviation. The baseline mean between the two groups was compared by independent sample *t*-test, the rate was compared by *Χ*^2^ test, and the data not conforming to normal distribution were compared by Wilcoxon sign rank-sum test. Pearson's correlation analysis was used to assess the relative expression levels of CXCL3 and CEA in plasma and CRC tissues. The CRC diagnostic value of plasma CXCL3 was estimated by receiver operating characteristic curve analysis (ROC). *P* < 0.05 was considered to be statistically significant.

## 3. Results

### 3.1. CXCL3 Was Highly Expressed in CRC Tissues

**Figure 1:**
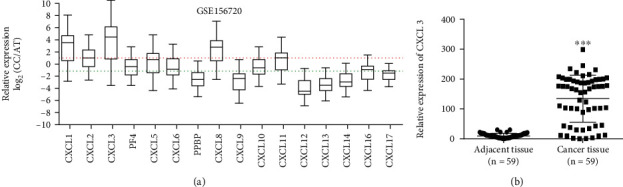
(a) Relative mRNA expression of CXCLs in CRC compared to adjacent non-tumor tissues (AT) in the GSE156720 dataset. CXCL1, CXCL2, CXCL3, CXCL5, CXCL8, and CXCL12 were highly expressed in CRC tissue compared to adjacent nontumor tissue (AT) (mean log2 (CC/AT) ≥ 1 red line), while CXCL12, CXCL13, and CXCL14 were poorly expressed in CRC tissue (mean log2 (CC/AT) ≤ 1 green line). (b) qRT-PCR analysis of CXCL3 expression in CRC tissue (*n* = 59) compared to adjacent nontumor tissue (AT) (*n* = 59).

The relative mRNA expression of CXCLs in CRC was analyzed with the public GEO dataset, GSE156720. CXCL1, CXCL2, CXCL3, CXCL5, CXCL8, and CXCL12 were highly expressed in CRC tissue compared to adjacent nontumor tissue (AT) (mean log_2_ (CC/AT) ≥ 1), while CXCL12, CXCL13, and CXCL14 were poorly expressed in CRC tissue (mean log_2_ (CC/AT) ≤ 1). CXCL3 was the most enriched (mean log_2_ (CC/AT) = 3.96) (Figure 1(a)). The mRNA levels of CXCL3 in 59 pairs of CRC and adjacent tissues (AT) were examined by qRT-PCR. mRNA levels of CXCL3 were greater in CRC tissues compared to matched adjoining tissue (*P* < 0.001) Figure 1(b).

### 3.2. Preoperative Plasma CXCL3 Levels Are Elevated in CRC Patients

**Figure 2:**
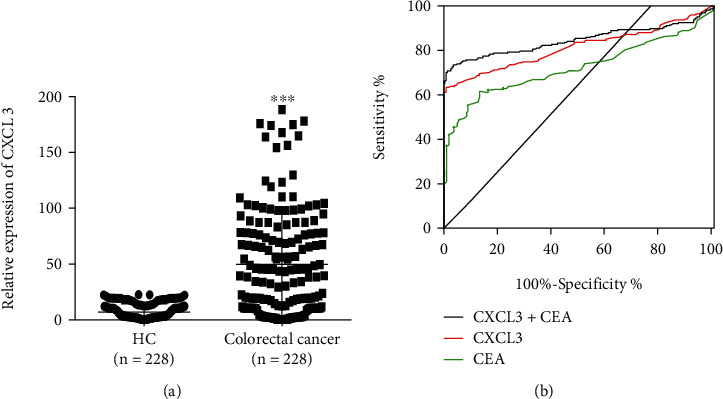
(a) qRT-PCR analysis was taken to explore the plasma CXCL3 levels for CRC patients and healthy controls (HC). (b) ROC curve analysis of the CRC diagnostic value of plasma CXCL3 levels.

228 patients diagnosed with CRC (included 59 surgical cases and 169 non-surgical cases and 219 healthy control subjects (HC)) were enrolled in this study. Plasma CXCL3 levels were measured for CRC patients and HC. Compared to HC (8.82 ± 6.79 pg/ml), preoperative plasma CXCL3 levels were significantly elevated (*P* < 0.001) for patients with CRC (71.15 ± 67.84 pg/ml, Figure 2(a)). To explore the diagnostic value of CXCL3, a ROC curve was constructed. The outcome showed an AUC of 0.81 (95% confidence interval (*CI*): 0.77~ 0.86) with a sensitivity of 0.71 and a specificity of 0.82, and the cutoff value of 15.5. When plasma CXCL3 and CEA values were combined for the diagnosis of CRC, the AUC increased to 0.85 (95% CI: 0.81~ 0.89), and the sensitivity and specificity were 0.74 and 0.96, respectively, indicating that CXCL3 added a significant advantage for a diagnosis of CRC (Figure 2(b) and Table 1).

### 3.3. Relationships among CXCL3 in CRC Plasma, CXCL3 in Cancer Tissue, and Plasma CEA

**Figure 3:**
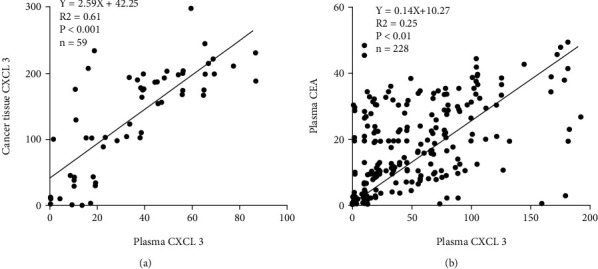
Pearson's correlation analysis was used to explore the relationship between CXCL3 in CRC plasma and CXCL3 in cancer tissue (a) and the relationship between CXCL3 and CEA in CRC plasma (b).

Pearson's correlation analysis showed that the level of CXCL3 in plasma of CRC patients was positively related to the expression of CXCL3 in cancer tissue (*r* = 0.78, *P* < 0.01), Figure 3(a). Further, the level of CXCL3 in plasma of CRC patients was positively related to plasma CEA (50.85 ± 46.93; *r* = 0.50, *P* < 0.01) (Figure 3(b)).

### 3.4. Relationships among Plasma CXCL3, CRC Clinicopathologic Features, and CRC Patient Survival Time

**Figure 4:**
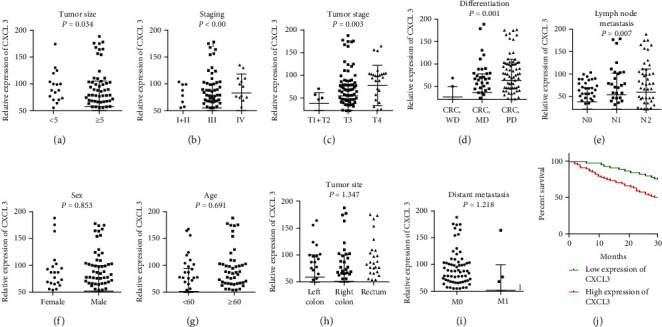
Associations among preoperative plasma CXCL3 levels and clinical parameters of CXC patients. (a) tumor size, (b) staging, (c) tumor stage, (d) differentiation, (e) lymph node metastasis, (f) sex, (g) age, (h) tumor site, (i) distant metastasis, and (j) Kaplan–Meier estimated disease-free survival in patients with high or low CXCL3 expression. Higher CXCL3 expression was associated with poorer disease-free survival (*P* < 0.01).

The patients with CRC were divided into high- and low-expression groups based on the cutoff value derived from the ROC curve. There were 94 cases in the increased expression group and 75 in the low expression group. The plasma expression of CXCL3 in CRC was related to many clinicopathological Characteristics (Table 2) such as tumor size (*P* = 0.034, Figure 4(a)), staging (*P* < 0.001, Figure 4(b)), tumor stage (*P* = 0.003, Figure 4(c)), differentiation (*P* = 0.001, Figure 4(d)), and lymph node metastasis (*P* = 0.007, Figure 4(e)), but not sex (*P* = 0.853, Figure 4(f)), age (*P* = 0.691, Figure 4(g)), tumor site (*P* = 1.347Figure 4(h)), or distant metastasis (*P* = 1.218, Figure 4(i)). To further assess the independently correlated clinical parameters of plasma CXCL3 levels, multivariate stepwise regression analysis was performed. When plasma CXCL3 levels were set as the dependent variable, and sex, age, tumor site, tumor size, staging, tumor stage, differentiation, lymph node metastasis, and distant metastasis were set as the independent variables, staging (standard *β* = 0.773, *P* = 0.042), tumor stage (standard *β* = 1.038, *P* = 0.038), and differentiation (standard *β* = 1.229, *P* = 0.024) were found to be independent factors of plasma CXCL3 levels (Table 3).

Kaplan–Meier analysis demonstrated an overall survival of patients in the high CXCL3 expression group to be shorter than in the low expression group (HR: 2.556, 95% CI: 1.306-4.223, *P* < 0.01, Figure 4(j)). Next, we used COX regression analysis to assess the correlation between plasma CXCL3 level and overall survival. The death events and follow-up time were the dependent variables; independently correlated clinical parameters such as sex, age, tumor site, tumor size, staging, tumor stage, differentiation, lymph node metastasis, distant metastasis, and plasma CXCL3 level were set as the independent variables; plasma CXCL3 level (HR: 2.017, 95% CI: 0.916-3.274, *P* < 0.01, Figure 4(j)) and tumor stage (HR: 1.809 95% CI: 0.726-2.612, *P* < 0.01) were found to be independent factors of overall survival.

## 4. Discussion

CRC is the third most malignant tumor worldwide. Diagnosis is typically at a middle or advanced stage, which is challenging to treat, resulting in a high degree of mortality. Studies have shown the incidence of CRC to be closely related to the composition of the diet. Unhealthy high fat or high carbohydrate western diets significantly increase the incidence of CRC [18]. Despite the wide range of screening programs available for clinical use, poor early detection of CRC remains a global problem [19, 20]. The internationally recognized laboratory diagnostic markers for CRC are CEA and CA19-9. However, the specificity and sensitivity of these markers do not meet current clinical need. Hence, identifying highly sensitive and specific diagnostic markers for the early diagnosis of CRC is essential.

CXCL3 is encoded by the human GRO gene, located within chromosomal region, 4q13.3. Alternative splicing results in multiple transcript variants [21]. CXCL3 is a member of the CXC chemokine subfamily, which is also identified as the growth regulated oncogene (GRO)*γ*. CXCL3 has many essential functions, including leukocyte chemotaxis [22], promotion of angiogenesis and tumor development [12], immune regulation [23], and interactions with other cytokines to form connections between inflammation, immune regulation, and tumorigenesis [24]. During inflammation, macrophages, mesothelial cells, and epithelial cells secrete CXCL3. These cells are the primary sources of CXCL3. CXCL3 interaction with its cognate receptor activates neutrophils to secrete cytokines that promote angiogenesis, one of the most essential steps in tumor development [25, 26]. Interaction of CXCL3 with its receptors not only induces the formation of new blood vessels but also facilitates the proliferation of tumors and the secretion of proteolytic enzymes, which disrupt the extracellular matrix and basement membrane, facilitating tumor invasiveness [27, 28]. CXCL3 levels are elevated in many tumors and immune diseases [29, 30], although there are no reports of CXCL3 expression in CRC.

This study demonstrated the expression of CXCL3 in CRC cancer tissue to be significantly higher than in matched adjacent tissue (*P* < 0.001). Compared to healthy controls, the plasma level of CXCL3 in patients with CRC was substantially higher (*P* < 0.01). Further, with an AUC of 0.81 (95% CI: 0.77~ 0.86) and a sensitivity of 0.71, and a specificity of 0.82, the ideal cutoff value was 15.5. When plasma CXCL3 and CEA values were combined for the diagnosis of CRC, the AUC increased to 0.85 (95% CI: 0.81~ 0.89) and the sensitivity and specificity to 0.74 and 0.96, respectively, indicating that CXCL3 was advantageous for the diagnosis of CRC. As such, a combination of CXCL3 and CEA was sensitive and specific for CRC diagnosis, providing reliable data for early clinical diagnosis of CRC. Spearman's rank correlation analysis showed that the level of CXCL3 in plasma of CRC patients was positively related to the expression of CXCL3 in cancer tissue (*r* = 0.78, *P* < 0.01). Further, the level of CXCL3 in plasma of CRC patients was positively related to CEA (*r* = 0.50), with the plasma expression of CXCL3 in CRC related to tumor size, staging, tumor stage, differentiation, and lymph node metastasis, but not to sex, age, tumor site, or distant metastasis. Kaplan–Meier analysis demonstrated overall survival of patients with high CXCL3 expression to be shorter than that of patients with low expression (*P* < 0.01).

There are limitations to this study. First, results may be confounded by variations in clinical case screening and differences in treatment. Second, cases were selected from only one hospital and may not represent other local or national sites. Third, the study was cross-sectional and requires confirmation of the role of CXCL3 in the pathogenesis of CRC cell differentiation, invasion, metastasis, clinical treatment, and prognosis. Such confirmation will be the purpose of our subsequent studies.

In summary, this study analyzed the expression of CXCL3 in cancer tissue and plasma of CRC patients and found relationships among CXCL3 and patient clinic pathological characteristics. Also, a potential clinical diagnostic value for the measurement of plasma CXCL3 in CRC patients was found. The area under the curve analysis allowed for a better understanding of the CRC diagnostic value of CXCL3. It is important to note that the onset of CRC is insidious and that patients are often referred to a doctor at an advanced disease stage, limiting treatment options. Therefore, there is an urgent need for an early diagnostic marker with high sensitivity and specificity as a means by which to improve clinical diagnosis. That need will be the focus of our future work.

## Figures and Tables

**Table 1:** ROC curve analysis of the diagnostic value of plasma CXCL3 levels for CRC patients.

Characteristics	AUC	SE	*P*	95% CI	Sen	Spe	Youden index
CXCL3 + CEA	0.85	0.04	<0.001	0.81~0.89	0.74	0.96	0.70
CXCL3	0.81	0.03	<0.001	0.77~0.86	0.71	0.82	0.53
CEA	0.72	0.05	<0.001	0.67~0.77	0.61	0.80	0.41

CXCL3: CXC-chemokine ligand 3; CEA: carcinoembryonic antigen; CRC: colorectal cancer.

**Table 2:** Associations among preoperative plasma CXCL3 levels and clinical parameters of CRC patients.

Clinicopathological characteristics	Total (*n* = 228)	Expression of CXCL3		*P* value
		High (*n* = 129)	Low (*n* = 99)	
Sex				0.853
Female	71	39	32	
Male	157	91	66	
Age (years)				0.691
≥ 60	155	88	67	
< 60	73	39	34	
Tumor site				1.347
Left colon	49	38	11	
Right colon	78	32	46	
Rectum	101	57	44	
Tumor size (cm)				0.034
≥ 5	142	74	68	
< 5	86	52	34	
Staging				<0.001
І + II	76	42	34	
III	132	75	57	
IV	20	11	9	
Tumor stage				0.003
T1 + T2	9	6	3	
T3	179	108	71	
T4	40 29	24	16	
Differentiation				0.001
CRC, WD	9	6	3	
CRC, MD	96	53	43	
CRC, PD	123	81	42	
Lymph node metastasis				0.007
N0	81	43	38	
N1	58	45	13	
N2	89	70	19	
Distant metastasis				1.218
M0	199	110	89	
M1	29	18	11	

CXCL3: CXC-chemokine ligand 3; TNM, tumor (T), nodes (N), and metastases (M). CRC: colorectal cancer; CRC WD: well-differentiated cancer; CRC MD: moderately differentiated cancer; CRC PD: poor-differentiated cancer.

**Table 3:** Multivariate stepwise regression analysis of plasma CXCL3 levels.

Independent variable	*β*	S.E.	*P* value	OR	95% CI
Staging	0.773	0.347	0.042	2.163	1.016-4.522
Tumor stage	1.038	0.441	0.038	0.306	0.139-0.995
Differentiation	1.229	0.572	0.024	3.227	1.185-7.952

Variables of the original model included: sex, age, tumor site, tumor size, staging, tumor stage, differentiation, lymph node metastasis, and distant metastasis.

## Data Availability

The data used to support the findings of this study are available from the corresponding authors upon request.
